# Supplementary material for: Red and Blue Light Induce Soybean Resistance to Soybean Mosaic Virus Infection through the Coordination of Salicylic Acid and Jasmonic Acid Defense Pathways
Source: Viruses. 2023 Dec 7;15(12):2389. doi: 10.3390/v15122389 (PMC10747522; doi:10.3390/v15122389)
Supplement: Supplementary file 1 [file viruses-15-02389-s001.zip › viruses-2669281-supplementary.pdf]

Supplementary Material

Red and Blue Light Induce Soybean Resistance to Soybean Mosaic Virus Infection through the Coordination of Salicylic Acid and Jasmonic Acid Defense Pathways

Jing Shang [1,*,†], Siqi Zhang [1,†], Junbo Du [1], Wenming Wang [2], Kai Li [3], Wenyu Yang [1]

1 Sichuan Engineering Research Center for Crop Strip Intercropping System and College of Agronomy, Sichuan Agricultural University, Chengdu 611130, China; shangjing_edu@163.com (J.S.); zhangsiqi_1215@163.com (S.Z.); junbodu@hotmail.com (J.D.); mssiyangwy@sicau.edu.cn (W.Y.)
2 State Key Laboratory of Crop Gene Exploration and Utilization in Southwest, Sichuan Agricultural University, Chengdu 611130, China; j316wenmingwang@163.com (W.W.)
3 National Center for Soybean Improvement, National Key Laboratory for Crop Genetics and Germplasm Enhancement, Key Laboratory of Biology and Genetic Improvement of Soybean, Ministry of Agriculture, Nanjing Agricultural University, Nanjing 210095, China; kail@njau.edu.cn (K.L.)
* Correspondence: shangjing_edu@163.com; Tel.: +86-028-86290872
† These authors contributed equally to this work.

Supplementary Table S1 Light quality conditions under different treatments.

Treatment	Control	Experiment
white light	WN	WS
red light（625±10）nm	RN	RS
blue light（465±10）nm	BN	BS
far-red light（735±10）nm	FRN	FRS

Note: WS, SMV infection under white light; WN, Normal under white light; RS, SMV infection under red light; RN, Normal under red light; BS, SMV infection under blue light; BN, Normal under blue light; FRS, SMV infection under far-red light; FRN, Normal under far-red light. Photosynthetic Photon Flux Density(PPFD)=380±10 $\mu mol \cdot m^{-2} \cdot s^{-1}$.

Supplementary Table S2 Primers used for identification of the virus and qRT-PCR assays.

Gene_ID	Forward primer （5'-3'）	Reverse primer （5'-3'）	Reference
SMV-CP	AGCTYGCTTCGTCTGGAAAA	AGGACAACAAACATTGCCGT	Zhao et al. [1]
SMV-CP	GCTTGGACCACTTGCT	ACACCCATCTGCTCATC	This study
Actin	CCATGTTCCCTGGTATTGCTG	GTATTTTCTCTCCGGTGGTGC	Zhao et al. [1]

Supplementary Table S3 Primers used in detection of defense gene expression levels.

Gene_ID	Forward primer （5'-3'）	Reverse primer （5'-3'）	Reference
100217338	CTGGTCTTTCTGCGTCAA	CGTAAGGGCTTCCATTCT	This study
100217339	GGATGACTTGCCTGACTT	GAGGGATGATGAGGATGA	This study
100807250	GCTGCGTATGCTCAAGAC	CACCACCAGAGTGGACAA	This study
100776311	GCGAGCTAAGAGCTGTAC	CTGAAGCCACCAATCCAC	This study
100795733	GCTTTAGGCTCGGTTCTC	GTGCTGTACGAGGTTCCA	This study
100779239	GCAAACTGAGGCAAAGAC	GCACCAACAGCGAAAATC	This study
100793081	AACAGAACGACCGTGGCA	GGCTCTTGAGGGAGTGGA	This study
100804965	CCGTCGTGGATCAAAGCG	AGCGTGGTGAACCTGAAACT	This study
100803653	AAATCAAGCACCACTCAA	CACCTCAGCCTGCAACTC	This study
100790598	ATGGAAAGCGACAGGAAT	TAGCGTCAACCGAAGAGG	This study
100805418	ATCTTTACTTTCAGGTGGTC	TTCAGCAGCCTCTTCTTC	This study
100816370	TTTATGTCTGTTTGTCGGTTAG	TTCCGTGTTGTTCTTTAGTTAG	This study
100786151	ATCATCTCAATCACCCTC	CACTCCCAAATACTCCTC	This study
102666009	ACTGTTACTCTTCTGGCTAC	GATACGAAGTTATGGAGG	This study
100816991	AACAGAACGACCGTGGCA	GGCTCTTGAGGGAGTGGA	This study

732589	CGCTCACTGCCATTGCTC	TGCGGATGACGGTGCTCT	This study

Supplementary Table S4 Primers used in detection of differentially expressed genes.

Gene_ID	Forward primer （5'-3'）	Reverse primer （5'-3'）	Reference
100127393	ACCTCCTGCTTCATCCAC	TGCTGTTACAAGGCGTCA	This study
100127397	CCTCAAGCTGCGAAGAGGAA	TCAGGAGTGGCAACCGAATC	This study
100127421	TTTATGTCTGTTTGTCGGTTAG	TTCCGTGTTGTTCTTTAGTTAG	This study
100816563	ATAGCAATGATGGGTGGT	ATCAAATCGTCCAAACAC	This study
547647	GCATAAATCAACCAGCGAAAG	TGGAACGTGGGAGGACAT	This study
100306363	GGAGCCATTGGAGAAGAT	TAGCCTTGGTGAAACCTG	This study
100805346	GCTGCGTATGCTCAAGAC	CACCACCAGAGTGGACAA	This study
100816824	CCTAAGTGGGAAAGATGC	TCCAAACGACCTGAGTGT	This study
100802408	GCGAGCTAAGAGCTGTAC	CTGAAGCCACCAATCCAC	This study
100792603	GGAGCCATTGGAGAAGAT	TAGCCTTGGTGAAACCTG	This study
100775870	GGATGACTTGCCTGACTT	GAGGGATGATGAGGATGA	This study
547787	CTGGTCTTTCTGCGTCAA	CGTAAGGGCTTCCATTCT	This study
778184	GCATAAATCAACCAGCGAAAG	TGGAACGTGGGAGGACAT	This study
100101837	ATCTTTACTTTCAGGTGGTC	TTCAGCAGCCTCTTCTTC	This study
732590	GGCTCTTTAGGGTTTATG	AGGTTGCTGGTTGTTATT	This study

Supplementary Table S5 Overlap analysis of differentially expressed genes in plant-pathogen interaction pathway under four light quality treatments.

Gene ID	Description	Log2FC WS/WN	Log2FC RS/RN	Log2FC BS/BN	Log2FC FRS/FRN

100794273	3-ketoacyl-CoA synthase 11	7.44	5.07	5.99	-2.33
100127397	WRKY transcription factor 62	5.62	6.34	5.19	1.89
100816563	putative calcium-binding protein CML19	4.77	8.68	11.64	-6.41
100127421	WRKY transcription factor 19	1.87	2.98	4.43	3.42
547787	calmodulin	1.31	3.43	2.74	2.22
100776837	probable WRKY transcription factor 33	2.49	4.26	3.39	-1.04
100801339	mitogen-activated protein kinase 3	-1.30	4.28	4.08	-1.44
100805346	disease resistance protein RPM1	3.45	6.12	7.54	-1.98
100775870	protein EDS1-like	3.82	2.97	2.19	-2.51
100791756	probable calcium-binding protein CML46	1.53	3.63	2.08	-2.79
100127393	WRKY transcription factor 49	2.49	4.11	3.69	-4.13

Supplementary Table S6 Non-overlapping Analysis of differentially expressed genes in Plant-pathogen interaction Pathway under four Light quality treatments.

Gene ID	Description	Log2FC WS/WN	Log2FC RS/RN	Log2FC BS/BN	Log2FC FRS/FRN
100813092	probable calcium-binding protein CML25	6.76	-	-	-
100807250	pathogenesis-related protein 1	6.64	7.28	-	-
100305637	uncharacterized 100305637	6.18	-	-	-
100795656	calmodulin-1	5.33	-	-	-
100816236	cyclic nucleotide-gated ion channel 1	4.48	-	-2.93	-
100801879	probable cyclic nucleotide-gated ion channel 14	4.05	-	4.12	-
112998383	putative calcium-binding protein CML19	-	9.98	14.41	-6.85

111240476	putative calcium-binding protein	-	8.05	8.71	-
100814475	calcium-dependent protein kinase 26	-	7.95	8.15	-
100812342	respiratory burst oxidase homolog protein B	-	6.7	5.87	-1.38
100806194	pathogenesis-related protein 1	-	6.63	-	-3.03
100305458	NBS-LRR type disease resistance protein	-	6.41	6.36	-1.58
100526868	uncharacterized 100526868	-	6.31	5.23	-
732590	WRKY transcription factor 51	2.38	4.90	-	-
100777554	protein SGT1 homolog B-like	-	4.75	2.11	-1.32
100783572	calcium-binding allergen Ole e 8	1.35	4.63	3.38	-
100789363	disease resistance protein RPM1	1.02	4.34		
547530	mitogen-activated protein kinase 1	-	4.34	5.04	-
100500636	calcium-binding EF-hand family protein	3.18	4.1	3.82	-
100815829	WRKY transcription factor 22	-	-7.22	-	-
100808554	RPM1-interacting protein 4	-	-7.65	-	-
100798531	putative calcium-binding protein CML19	-	-	10.50	-
100777057	putative calcium-binding protein CML19	-	-	9.30	-
100815308	putative cyclic nucleotide-gated ion channel 8	-	-	8.10	-
100820422	calcium-dependent protein kinase 34	-	-	7.32	-
100807117	calcium-binding protein CAST	-	-	5.94	-
100787013	calmodulin-like protein 30	1.52	-	5.41	-

100812778	probable calcium-binding protein CML46	-	-	4.96	-
100527117	putative calcium-binding protein	-	-	4.28	-
100820237	protein EDS1L	-	-	4.21	-2.08
100796922	calmodulin-like protein 3	-	-	4.17	-6.4
100776947	3-ketoacyl-CoA synthase 12	-	-	-	9.59
100792610	calcium-dependent protein kinase 2	3.33	-	-	7.47
100809149	3-ketoacyl-CoA synthase 12	-	-	-	4.96

Supplementary Table S7 Analysis of key differentially expressed genes in SA, JA and ET signal transduction pathways under four light quality treatments.

Gene ID	Description	Log2FC WS/WN	Log2FC RS/RN	Log2FC BS/BN	Log2FC FRS/FRN
Key genes of the SA signal transduction pathway					
100807250	Pathogenesis-related protein 1	6.83	6.43	7.39	-
100806194	Pathogenesis-related protein 1	7.65	4.78	5.32	-6.71
100776311	bZIP transcription factor 3（TGA 3）	1.07	3.78	-0.25	1.11
100217339	NPR1-2 protein	3.47	4.23	1.78	3.40
100217338	NPR1-1 protein	0.96	3.29	2.33	2.82
Key genes of the JA signal transduction pathway					
100795733	Transcription factor MYC2	1.71	3.46	5.91	-3.44
100777944	Jasmonate-amino acid synthetase JAR1	-2.01	-0.96	-2.73	-3.34

100804965	Transcription factor MYC2	2.94	2.69	10.21	-7.14
100792358	Jasmonate-amino acid synthetase JAR1	-1.71	-2.26	0.91	0.84
100306655	Jasmonate ZIM domain-containing protein 12	-4.53	-2.05	0.86	-1.64
100793081	Coronatine-insensitive protein 1-like	-1.38	2.50	-0.62	-5.33
Key genes of the ET signal transduction pathway					
100816370	Ethylene-responsive transcription factor ERF113	-0.51	1.61	1.13	-2.47
100805418	EIN3-binding F-box protein 1	6.55	3.24	1.95	3.54
100790598	Ethylene-responsive transcription factor 1B	3.02	2.13	2.26	1.37
100804481	Ethylene-responsive transcription factor 1B	2.87	2.82	7.23	3.03
100792946	Ethylene-responsive transcription factor ERF112	2.07	1.94	6.34	4.27
100813781	Ethylene-responsive transcription factor ERF024	3.94	1.24	3.75	1.89
100785364	Ethylene-responsive transcription factor ERF061	2.66	-1.50	3.27	7.36
100803653	Ethylene-responsive transcription factor 1A	3.46	2.81	2.25	-1.07

Reference

1 Zhao, Q.; Li, H.; Sun, H.; Li, A.; Liu, S.; Yu, R.; Cui, X.; Zhang, D.; Wuriyanghan, H. Salicylic acid and broad spectrum of NBS-LRR family genes are involved in SMV-soybean interactions. *Plant Physiol. Biochem.* **2018**, *123*, 132–140.
